# Anti-Inflammation and Protective Effects of *Anethum graveolens* L. (Dill Seeds) on Esophageal Mucosa Damages in Reflux Esophagitis-Induced Rats

**DOI:** 10.3390/foods10102500

**Published:** 2021-10-18

**Authors:** Hyeon-Hwa Nam, Li Nan, Byung-Kil Choo

**Affiliations:** 1 Herbal Medicine Resources Research Center, Korea Institute of Oriental Medicine, Naju-si 58245, Korea; namhh@kiom.re.kr; 2 Agricultural College, Yanbian University, Yanji 133002, China; nanli12@163.com; 3 Department of Crop Science & Biotechnology, Jeonbuk National University, Jeonju 54896, Korea

**Keywords:** *Anethum graveolens* L., anti-inflammation, NF-κB, reflux esophagitis

## Abstract

*Anethum graveolens* L. (dill seeds) are important medicinal and functional foods in Europe and central and south Asia, often used as a seasoning in daily diets. *Anethum graveolens* L. seeds (AGS) are used to treat indigestion and have shown physiological activities such as those against hypoglycemia and gastroesophageal disease. This study explored the protective effects of AGS extract on mucosal damages and inflammation in reflux esophagitis rats. AGS inhibited cellular inflammation including NO production and the expression of inflammatory proteins (iNOS and COX2 etc.), cytokines (IL-1β and TNF-α) and nuclear transfer factor related to NF-κB signaling caused by LPS stimulation in vitro. Furthermore, reflux esophagitis-induced rats were used to observe the anti-inflammatory effect of AGS. Tissue staining and inflammation-related protein expression of rats with acute reflux esophagitis indicated that AGS improved this inflammatory response, such as COX-2 and TNF-α in mucosa. In conclusion, AGS have good physiological activity and the possibility of being used as a medicinal food and a functional resource for the prevention and therapy of gastroesophageal diseases.

## 1. Introduction

*Anethum graveolens* L. seeds (AGS), which belong to the Umbeliferae family, have a strong spicy odor and an acrid taste and have been used as medicine, spices and for aromatic plants since ancient times [1,2,3]. This herb is used to treat stomachache and indigestion, as well as dyspepsia and gastrointestinal diseases [4,5]. In addition, this herb is extensively used in the food industry as a flavoring agent, including in Indian curry, ham, and other meat products [6,7,8]. The physiologically active constituents of AGS include tannins, limonene, Keaton, carvon and fatty acids and it is predicted that limonene, carvon, chlorogenic acid, and linoleic acid exhibit anti-inflammation and gastroprotection effects [9,10,11,12,13]. The gastroprotection effect of limonene was demonstrated by increased mucus production and anti-inflammatory response in rats. According to research focused on AGS, it shows hypolipidemic, hypoglycemic, and antibacterial bioactivity [12,13,14,15]. Although AGS have been used to treat gastrointestinal diseases, related studies have not investigated its involvement in anti-inflammation and gastroprotective effects.

Inflammation, a natural defense, is a response to harmful external stimulation such as physical damage, ultraviolet radiation, or pathogen invasion [14,16]. Various pro-inflammatory cytokines are secreted and produce a series of symptoms including redness, fever, and pain [17]. These symptoms are relieved with a reduction of inflammation. However, if the inflammatory reaction is too intense or prolonged, it can lead to tissue damage, serious disease, and even cancer [18]. Therefore, inhibiting its overgeneration is an important step in the treatment of various inflammatory diseases.

Reflux esophagitis is one of the gastroesophageal reflux diseases that seriously affects patient life quality around the world [19]. This disease is receiving growing attention due to its increasing incidence and declining age of onset. Its occurrence is often caused by poor eating habits, smoking, or stress [20,21]. Destruction of the lower esophageal sphincter causes the stomach contents to flow back into the esophagus, creating inflammatory lesions that can lead to esophageal erosion and even esophageal cancer [22]. The main drugs used to treat inflammation are nonsteroidal anti-inflammatory drugs (NSAIDs). These drugs have anti-inflammatory, anti-rheumatic, analgesic, and antipyretic effects and are used clinically for symptoms such as rheumatoid arthritis, fever, and pain [23]. Although NSAIDs have good anti-inflammatory effects, long-term use can cause gastrointestinal dyspepsia, liver damage, allergies, and other adverse reactions [24]. Therefore, research is exploring natural plant sources that have good anti-inflammatory effects and no or few side effects [25]. To utilize the benefits of AGS for the digestive system and categorize it as a medicinal and functional food, research on its efficacy in digestive disorders is needed. This study demonstrated the therapeutic effects of AGS in reflux esophagitis-induced rats and the possible mechanisms of the responses.

## 2. Materials and Methods

### 2.1. Extraction

The AGS used in this study were purchased from Omniherb Company (Yakryeong-ro, Dongdaemun-gu, Seoul, Korea). The seeds were ground into powder with a mixer, 100% ethanol was added at a ratio of 1:10, and a multi heating mantle was used for three distillation extractions for 2 h each. The AGS extract was concentrated by a vacuum evaporator, lyophilized using a freeze-dryer, and stored at −20 °C until use. The 100 μg/mL of the AGS extracts were dissolved in DMSO as a stock solution, and were then used as a diluent in culture media for cell treatments. 

### 2.2. Chemical for HPLC Analysis

Three iridoids, two phenylpropanoids, and one triterpenoid were detected in a 70% ethanolic extract of *A. graveolens* using HPLC-ELSD. Chlorogenic acid (≥98) was purchased from KOC Biotech Corporation (Daejeon, Korea). Linoleic acid and elaidic acid were obtained from Aligent (Santa Clara, CA, USA) HPLC grade acetonitrile, ethanol, and distilled water were purchased from Merck (Darmstadt, Germany).

### 2.3. HPLC Analysis of Anethum graveolens L. Seeds (AGS)

Briefly, 16.63 mg of AGS was dissolved in 1 mL of 100% ethanol, and filtered through a syringe filter. Three standards were prepared as stock solutions in 30% acetone in methanol. HPLC analysis was performed on a chromatographic system equipped with a separation module (e2695), a 2424 evaporative light-scattering detector (ELSD; Waters Co., Milford, MA, USA), and nitrogen gas generator (Genius, Peak Scientific Instruments Ltd., Scotland, UK). Confirmations of peaks in AGS chromatograms were processed using the Empower 3 program (Waters Co., Milford, MA, USA). Liquid chromatography separations were performed on a Luna C8(2) column (5 μm, 4.6 × 250 mm, Phenomenex Inc. (Torrance, CA, USA)) with 10 μL injected volume and 0.9 mL/min flow rate. The mobile phase consisted of 0.05% formic acid in distilled water (A) and acetonitrile (B), and the linear gradient program was as follows: 100% A→80% A (5 min), 50% A→45% A (15 min), 45% A→30% A (17 min), 30% A→28% A (20 min), 28% A→7% A (40 min), 7% A→0% A (42 min), 0% A isocratic mode for 47 min. ELSD conditions were 50 of detector gain, 40 psi of nitrogen gas, and 50 °C of drift tube. All standard compounds were confirmed by comparing the retention time and λ_max_ of each compounds.

### 2.4. Cell Culture

Mouse mononuclear macrophage RAW264.7 cells were purchased from the Korea Cell Line Bank (Cancer Research Institute, Seoul National University, Chongno-gu, Seoul, Korea). Cells were cultured in 10% FBS medium with 100 units/mL penicillin and 100 μg/mL streptomycin (Welgene, Namcheon-ro, Seoul, Korea) at 37 °C and 5% CO_2_ in an incubator with sufficient humidity. 

### 2.5. Cell Cytotoxicity and Morphological Changes

Cells at a concentration of 5 × 10^5^ cells/mL in 100 μL of medium were seeded in a 96-well plate and treated with AGS (12.5, 25 and 50 μg/mL), chlorogenic acid, linoleic acid, and elaidic acid (3.13, 6.25, 12.5 μM) and LPS (1 μg/mL) (Sigma, St. Louis, MO, USA) for 18 h. The cytotoxicity of AGS on RAW264.7 cells was determined using EZ-Cytox according to the technical manual. Cell morphology was observed using a microscope at 40×. 

### 2.6. Nitric Oxide (NO) Production

Cells at a concentration of 5 × 10^5^ cells/mL in 100 μL of medium were seeded in a 96-well plate, pre-treated with AGS (12.5, 25 and 50 μg/mL), chlorogenic acid, linoleic acid, and elaidic acid (3.13, 6.25, 12.5 μM) for 1 h, and then induced by LPS (1 μg/mL) for another 18 h. The nitrite concentration in the cell culture medium was detected using the Griess method. Absorbance was measured with a multi-plate reader at 540 nm. The nitrite content was calculated using the standard curve created. 

### 2.7. Inflammatory Cytokines Analysis 

The amounts of TNF-α and IL-1β (R&D Systems, Minneapolis, MN, USA) in cell culture medium were measured using TNF-α and IL-1β/IL-1F2 immunoassay kits, respectively, according to the technical manual.

### 2.8. Inflammatory Proteins Analysis

Cells at a concentration of 1 × 10^6^ cells/mL in 2 mL of medium were seeded in a 6-well plate and were pre-treated with AGS (25 and 50 μg/mL) for 1 h. LPS (1 μg/mL) was then added to cells for 1 h and 18 h. Cells were lysed with the lysis buffer used in our previous research. Proteins were collected and quantified with protein assay reagent to prepare a loading sample. The appropriate gel percentage for electrophoretic separation was selected according to protein size. The separated proteins were transferred to PVDF membranes and blocked with 5% skim milk at room temperature for 2 h. The primary antibodies of iNOS, COX-2, NF-κB p65, p-NF-κB p65, IκBα, p-IκBα, and β-actin in PBST at a ratio of 1:1000 were added to the membrane and were incubated at 4 °C overnight. Cells were incubated with secondary antibodies (1:10,000) at room temperature for 2 h. Protein bands were observed using Bio-Rad imaging software.

### 2.9. Immunofluorescence Assay

Cells at a concentration of 2 × 10^5^ cells/mL in 2 mL of medium were plated in a 6-well plate with a 0.17 mm coverslip. Cells were pre-treated with AGS 50 μg/mL for 1 h. To activate the NF-κB signaling pathway, LPS (1 μg/mL) was added to cells and incubated for 30 min. Cells were fixed in 4% paraformaldehyde, and the cell membrane was destroyed by 0.5% Triton X-100 to allow antibodies to enter the nucleus. Antibodies were then blocked with 5% BSA containing 0.1% Triton X-100. Cells were incubated overnight at 4 °C with an NF-κB p65 primary antibody (Santa Cruz Biotechnology, Delaware Ave, Dallas, CA, USA) and 1% BSA (1:200). Cells were incubated with a secondary antibody (Santa Cruz Biotechnology, Delaware Ave, Dallas, CA, USA) and 1% BSA (1:1000) at room temperature in the dark for 2 h. Finally, cell nuclear staining and mounting were conducted using histology mounting medium with DAPI (Sigma-Aldrich, St. Louis, MO, USA). 

### 2.10. Animal Management

SD rats (7 weeks old and 200–220 g) were purchased from Hanil Experimental Animal Center (Samrye-ro, Wanju-gun, Jeonbuk, Korea). Rats were acclimated for one week in animal-specific breeding rooms with sufficient food and water. All experiments and the management of rats in this study were performed at the Jeonbuk National University in accordance with the regulations of the Animal Care and Use Committee of the Laboratory Animal Center of Jeonbuk National University (IACUC; CBNU 2020-011, date of approval, 17 February 2020). 

### 2.11. Reflux Esophagitis Rat Model

At the end of the adaptation period, rats were randomly divided into four groups (*n* = 8). In group 1, the normal control group (Normal), rats did not undergo drug pretreatment or surgery to induce esophageal inflammation and injury. In group 2, the esophageal inflammation and injury control group (RE control), rats were pre-treated with physiological saline by gavage 2 h before the surgery. In group 3, the ranitidine treatment group, rats were pre-treated with ranitidine (50 mg/kg) by gavage 2 h before the surgery; and in group 4, the AGS treatment group, rats were pre-treated with AGS (75 mg/kg) by gavage 2 h before the surgery. Esophageal inflammation and injury were induced under respiratory anesthesia by ligating the pylorus and the junction of the forestomach and stomach body to cause repeated reflux of gastric contents to the esophagus to induce esophageal inflammation and injury. After 4.5 h, all rats were anesthetized with ifran gas by a small animal anesthesia system, and the stomach and esophageal tissues were removed for analysis.

### 2.12. Ratio of Esophageal Mucosa Damages

The rat esophagus was cut longitudinally to expose the internal damage, and images were collected using a digital camera. The proportion of esophageal injury was analyzed using the Image J program. The calculation formula was the ratio of esophageal injury (%) = area of esophageal injury/total area of esophagus, as used in our previous study. 

### 2.13. Morphological Analysis in Esophgeal Mucosa

Esophageal tissue was fixed with 4% PFA, dehydrated, cleared, and prepared as a paraffin block. The tissue embedded in the paraffin block was cut into 5 μm sections and stained with hematoxylin and eosin. Tissue morphology was observed using a Leica microscope.

### 2.14. Extraction of Esophageal Tissue Proteins

Esophageal tissue was ground into a homogenate with a cold cytoplasmic protein lysis buffer. The tube was placed on ice for 30 min and then centrifuged at 3000 rpm for 3 min. Supernatant with cytoplasmic protein was collected. The nuclear protein lysis buffer was added to re-suspend the pellet, and the solution was placed on ice for 30 min and then centrifuged at 13,000 rpm for 10 min. Supernatant with nuclear protein was collected. The cytoplasmic and nuclear proteins were stored at −80 °C until use.

### 2.15. Statistical Analysis

Data in this study are expressed as mean ± SD. One-way analysis of variance (ANOVA) and LSD multiple post-hoc comparisons were carried out by SPSS 12.0 K. *p* values < 0.05 were considered significant.

## 3. Results

### 3.1. Cell Morphological Changes

**Figure 1:**
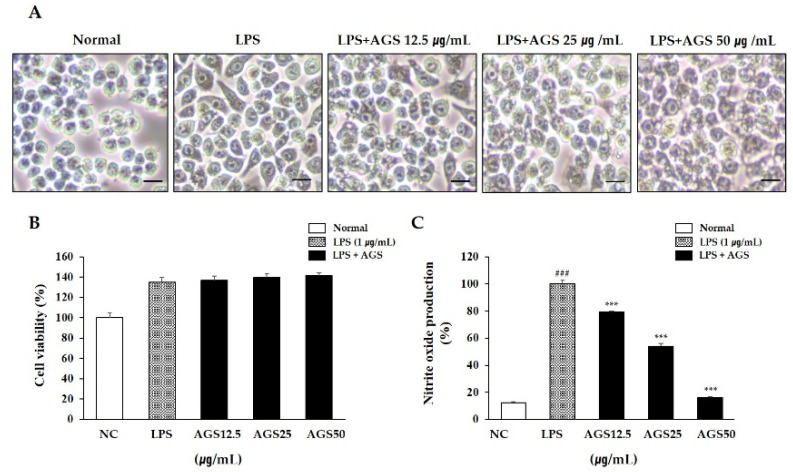
Effects of *Anethum graveolens* L. seeds (AGS) in cell cytotoxicity and nitrite oxide (NO) production. Cells treated with the AGS (12.5, 25, 50 μg/mL) and LPS (1 μg/mL) for 18 h. Effect of AGS on morphological transformation (**A**), cell viability (**B**) and production of NO (**C**) in LPS-induced RAW264.7 cells. Scale bar was 200 μm. Data were expressed as mean ± SD of duplicate experiment. Statistical analysis was performed for LPS compared with normal cells (^###^ *p* < 0.001) and each sample compared with LPS-induced cells (*** *p* < 0.001).

Treatment with LPS not only causes cell morphology change into an irregular shape with unclear cell boundaries, but also induces inflammation and tissue damage. In this study, activation and cell morphology changes were induced by LPS compared with the normal group, while AGS treatment prevented these morphological changes (Figure 1A). 

### 3.2. Cytotoxicity and Nitrite Oxide (NO) Production

Determining whether the extract has cytotoxicity is the basis for subsequent experimental research. In this study, a cytotoxicity assay kit was used to determine the effect of AGS on the viability of RAW264.7 cells (Figure 1B). In the concentration range of 12.5–50 μg/mL, the cells in the AGS-treated group maintained high cell activity compared to the normal group, indicating the safety of AGS in RAW264.7 cells. When an inflammatory reaction occurs in a cell, the cell secretes excessive amounts of NO, inflammatory mediators that have an important relationship with the development and exacerbation of inflammation. In this study, NO production is significantly increased in only the LPS treatment group compared to the normal group. However, as a result of treatment with AGS, NO production was dose-dependently decreased, and more than 50% scavenging activity was exhibited at a concentration of 50 μg/mL compared with the LPS control group (Figure 1C).

### 3.3. Inflammatory Cytokines Production

**Figure 2:**
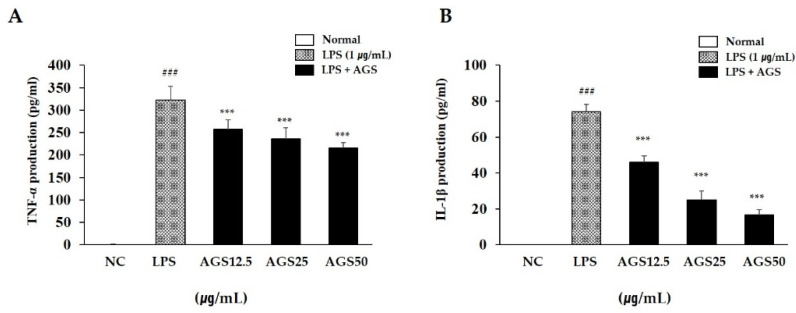
Inhibition effect of *Anethum graveolens* L. seeds (AGS) on cytokines production. Inhibition effect of AGS on production of TNF-α (**A**) and IL-1β (**B**) in LPS-induced RAW264.7 cells. Cells treated with the AGS (12.5, 25, 50 μg/mL) and LPS (1 μg/mL) for 18h. Data were expressed as mean ± SD of duplicate experiment. Statistical analysis was performed for LPS compared with normal cells (^###^ *p* < 0.001) and each sample compared with LPS-induced cells (*** *p* < 0.001).

When an inflammatory reaction occurs in a cell, the cell secretes excessive amounts of TNF-α and IL-1β. These cytokines are closely related to the occurrence and development of inflammation. In this study, LPS stimulation increased TNF-α and IL-1β production compared with normal control. AGS pretreatment significantly inhibited the production of these substances, confirming that AGS effectively inhibits LPS-induced cell inflammation (Figure 2).

### 3.4. Expression of Inflammatory Proteins

**Figure 3:**
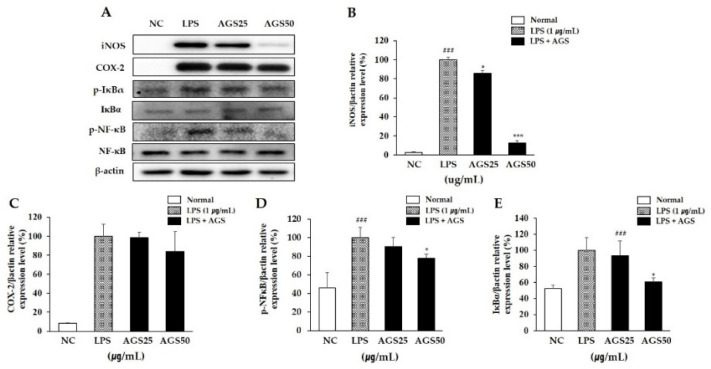
Inhibition effect of *Anethum graveolens* L. seeds (AGS) on expression of inflammatory proteins. Inhibition effect of AGS on expression level (**A**) of iNOS (**B**), COX-2 (**C**), p-NF-κB (**D**) and p-IκBα (**E**) proteins in LPS-induced RAW264.7 cells. Cells treated with the AGS (12.5, 25, 50 μg/mL) and LPS (1 μg/mL) for 1h and 18h. Data were expressed as mean ± SD of duplicate experiment. Statistical analysis was performed for LPS compared with normal cells (^###^ *p* < 0.001) and each sample compared with LPS-induced cells (* *p* < 0.05 and *** *p* < 0.001).

A sudden increase of the inflammatory proteins iNOS and COX-2 is an important signal for cells to activate an inflammatory response. Therefore, inhibiting the expression of both can prevent further inflammatory responses. In this study, the expression levels of iNOS and COX-2 increased sharply after LPS stimulation. With AGS pretreatment, the increase in expression was inhibited significantly, especially at a AGS concentration of 50 μg/mL (Figure 3A–C).

### 3.5. Phosphorylation of NF-kB and Ikba

The NF-κB signaling pathway regulates the expression of inflammatory proteins and pro-inflammatory cytokines. Therefore, inhibiting the activation of NF-κB can suppress inflammation. In this study, LPS induced an increase in the phosphorylation of IκBα and NF-κB and in the transfer of NF-κB into the nucleus. This suggests that the NF-κB signaling pathway was activated to regulate cellular inflammation. AGS pretreatment significantly suppressed the activation of NF-κB by inhibiting the phosphorylation of IκBα (Figure 3A,D,E). These results indicate that AGS inhibits cell inflammation induced by LPS through regulation of the NF-κB signaling pathway. 

### 3.6. Immunofluorescensce Assay of NF-kB Nuclear Transfer

**Figure 4:**
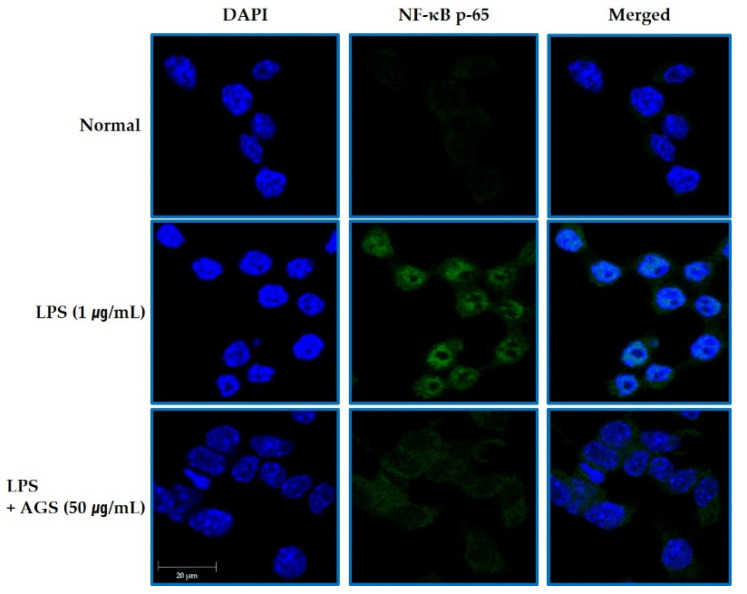
Inhibition the NF-kB Translocation by AGS. Inhibition effect of AGS nuclear transfer of NF-κB in LPS-induced RAW264.7 cells. Cells treated with the AGS (12.5, 25, 50 μg/mL) and LPS (1 μg/mL) for 1 h.

As shown in Figure 4, NF-κB was expressed mostly in the cytosol in normal control cells. In the LPS-induced cells, NF-κB translocated into the nucleus, which was markedly reduced with AGS. 

### 3.7. Esophageal Mucosal Damages Induced by Gastric Contents Reflux

**Figure 5:**
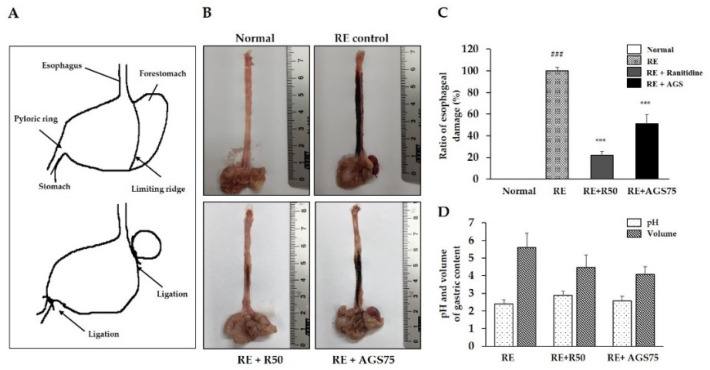
Observation of the esophageal mucosa damages. The sketch map of surgery of the reflux esophagitis rat model (**A**), representative microscopic image of the esophageal mucosa damage (**B**), ratio of esophageal damage (**C**), pH and volume of gastric contents (**D**), of the esophageal mucosa. Data were expressed as mean ± SD of duplicate experiment. Statistical analysis was performed for LPS compared with normal cells (^###^ *p* < 0.001) and each sample compared with LPS-induced cells (*** *p* < 0.001).

Gastric content reflux is one of the main features of reflux esophagitis. Repeated reflux causes esophageal mucosal damage and inflammation. In our study, continuous reflux of gastric contents induced by surgery led to extensive damage (including redness and hemorrhagic erosion) to the rats’ esophagi (Figure 5). However, gavage with ranitidine (positive control drug, 50 mg/kg) and AGS (75 mg/kg) 2 h before surgery effectively reduced esophageal tissue damages such as epithelial tissue destruction, redness and hemorrhage. Furthermore, AGS pre-treatment increased the pH of gastric contents and inhibited the secretion of gastric acid in RE rats. 

### 3.8. Histological Changes

**Figure 6:**
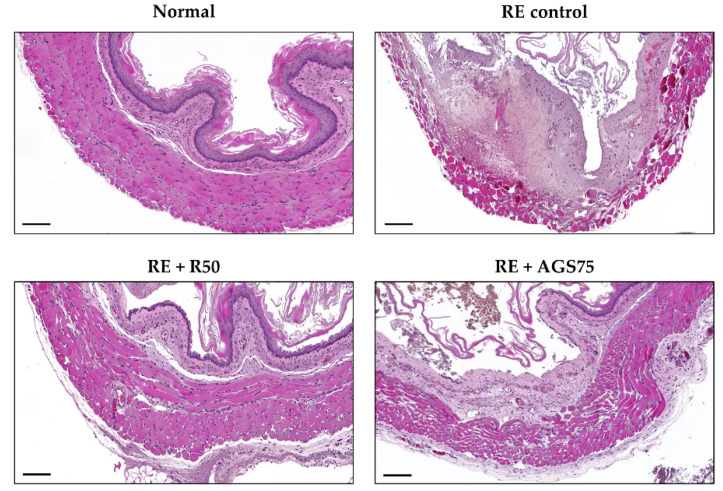
The histological observation on esophageal mucosa. Scale bar was 200 μm.

Histological observation is an important detection method for judging the integrity of tissue structure and the occurrence of tissue lesions. In this study, we observed a complete, tight, and clear esophageal tissue structure in the normal group (Figure 6). However, after gastric reflux, serious esophageal tissue lesions occurred, including tissue structure deformation, reduced tightness, and epithelial cell shedding. The lesions of esophageal tissue treated with ranitidine and AGS were greatly improved compared with RE control.

### 3.9. Expression of Inflammatory Proteins in Esophageal Mucosa

**Figure 7:**
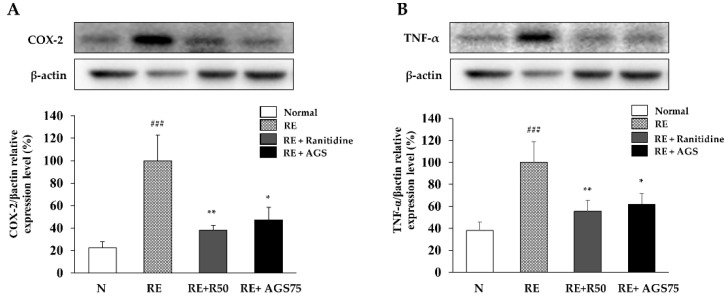
Expression of inflammatory proteins in esophagus. The expression of COX-2 (**A**) and TNF-α (**B**) in esophageal tissue. Data were expressed as mean ± SD of duplicate experiment. Statistical analysis was performed for LPS compared with normal cells (^###^ *p* < 0.001) and each sample compared with LPS-induced cells (* *p* < 0.05 and ** *p* < 0.01).

As an inducible enzyme, COX-2 is involved in regulating inflammation. TNF-α also play an important role in the inflammation process. Therefore, inhibiting the expression of COX-2 and TNF-α is one of the important factors that mediates the inflammatory response. In this study, repeated esophageal reflux caused esophageal inflammation and induced an increase in the expression of COX-2 and TNF-α, while ranitidine and AGS gavage treatment significantly inhibited this expression in esophageal tissue (Figure 7). 

### 3.10. Phytochemistry and Functional Properties of Anethum graveolens L. Seeds (AGS)

**Figure 8:**
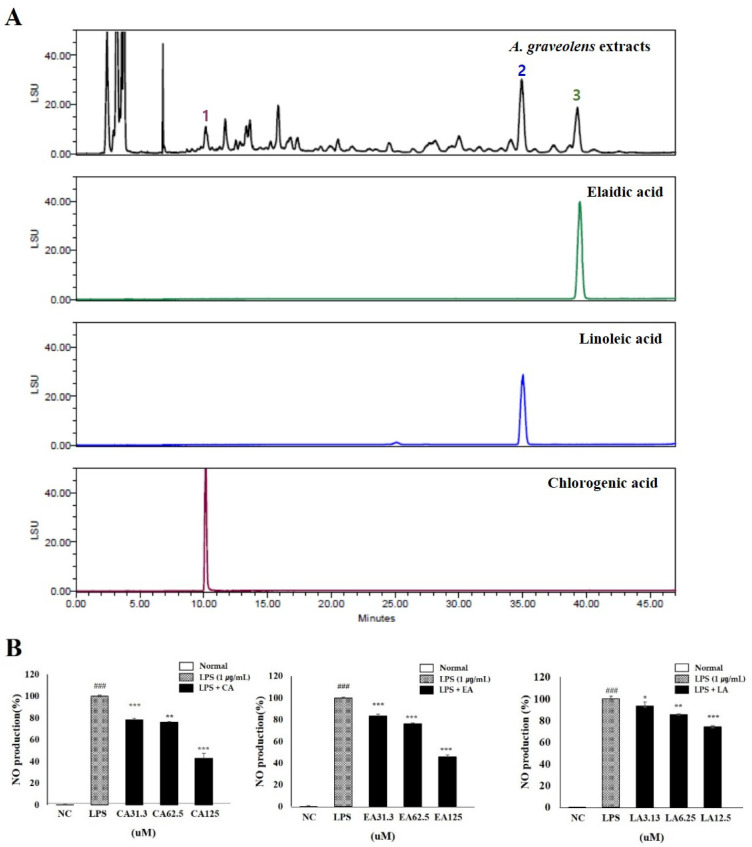
Phytochemistry and functional properties of *Anethum graveolens* seeds (AGS). Chromatogram of 70% ethanolic extract of AGS (**A**); chlorogenic acid at 10.2 min, linoleic acid at 35.1 min, and elaidic acid at 39.5 min using HPLC-ELSD and Luna C8 (2) column. Anti-inflammatory effects of chlorogenic acid (CA), linoleic acid (LA), and elaidic acid (EA) in LPS-induced RAW 264.7 cells (**B**). Cells treated with the chlorogenic acid and elaidic acid (31.3, 62.5, 125 μM), linoleic acid (3.13, 6.25, 12.5 μM), and LPS (1 μg/mL) for 18h. Data were expressed as mean ± SD of duplicate experiment. Statistical analysis was performed for LPS compared with normal cells (^###^ *p* < 0.001) and each sample compared with LPS-induced cells (* *p* < 0.05, ** *p* < 0.01 and *** *p* < 0.001).

Linoleic acid and elaidic acid has mainly been performed by GC with derivatization into methyl esters; however, the HPLC-ELSD method does not require derivatization [26]. Chlorogenic acid, linoleic acid, and elaidic acid were detected at approximately 10.157, 35.043, and 39.483 min, respectively. Amounts of three compounds were 11.805 ± 0.409, 30.957 ± 0.625, and 16.868 ± 0.158 μg/mg, respectively (Figure 8A). In order to confirm the inhibitory activity of the components on inflammation, the amount of NO produced in the RAW264.7 cells induced in inflammation was measured. The inhibitory effects of chlorogenic acid and elaidic acid were 57% and 54% in 125 μM, respectively. It was also confirmed that NO production was reduced by more than 25% at a concentration of 12.5 μM by linoleic acid treatment and confirming that linoleic acid is the highest anti-inflammatory effects (Figure 8B).

## 4. Discussion

Reflux esophagitis is a disease that requires long-term medication and has a high recurrence rate. Both its incidence area and rate are increasing [27]. Because phytomedicine has the advantages of good curative outcomes and fewer side effects, recent trials to improve digestive disorder therapy have focused on traditional herbal medicine and functional foods. AGS is commonly used in foods and is a well-known traditional medicine for several diseases of the stomach, liver, kidney, and brain [11]. To further explore the physiological activity of AGS, we determined its anti-inflammatory activity and protection effects on esophageal mucosal damages in vitro and in vivo. 

The LPS-induced macrophage RAW264.7 inflammatory model is commonly used to detect the anti-inflammatory activity of natural drugs [28,29]. RWA264.7 cells are also being used to research gastrointestinal mucosal damage and anti-inflammation mechanisms [30,31]. LPS, an endotoxin, transmits stimulation signals to cells by binding to intracellular functional LPS transmembrane receptors (Toll-like receptors), activating macrophages that cause the release of inflammatory mediators NO, TNF-α, and IL-1β and leading to inflammation and tissue damage [32]. It plays a very important biological role in cardio-cerebrovascular, nerve, and immune regulation. Among inflammation mediators, NO produced by iNOS is considered one of the major problems [33]. We identified the anti-inflammation of AGS by inflammation response in LPS-induced RAW264.7 cells. As shown in Figure 1, NO produced by LPS stimulation was significantly inhibited without cytotoxicity by treatment AGS extracts. The expression of pro-inflammatory cytokines TNF-α and IL-1β were also increased by LPS treatment but decreased by AGS treatment in a dose dependent manner. Previous studies have shown that the expression of inflammatory proteins and pro-inflammatory cytokines is mediated by the NF-κB signaling pathway [34,35]. When an inflammatory reaction occurs in a cell, the inhibitory protein IκBα is phosphorylated to release NF-κB, which then undergoes nuclear transfer to regulate the expression of various inflammation-related genes such as cytokines, iNOS and COX-2 [36]. Therefore, if IκBα can effectively inhibit the activation of NF-κB, the occurrence and exacerbation of the inflammatory response can be suppressed. The result showed that AGS effectively inhibited the expression of iNOS, COX-2, IκBα, and NF-κB and decreased translocation of the NF-κB into the nucleus in LPS-induced RAW 264.7 cells. In the present study, we demonstrated that AGS inhibited inflammation reaction by down-regulating pro-inflammatory mediators and suppressing the NF-κB signaling pathway.

Reflux esophagitis is induced by refluxed gastric acid and pepsin on esophageal epithelial cells. Chemical effects of refluxed gastric acid and pepsin lead to the acute inflammatory response and the death of surface epithelial cells in the esophagus [37,38]. According to several studies, to demonstrate the therapeutic effects of AGS on mucosal damages, we induced gastric content reflux to the esophagus by ligating the pylorus and the junction of the forestomach and stomach body [39]. Surgical induction of reflux produced apparent injury to the surface of the esophageal mucosa, including epithelial tissue destruction, redness and hemorrhage, compared to the normal group. However, these esophageal damages, such as redness and hemorrhagic lesions, were improved by about 50% in AGS 75 mg/kg groups. These visual symptoms are involved in the development of histopathological changes observed in esophageal mucosa. Our observations of histological changes to understand mucosal damages show that reflux esophagitis in this rat model involved the destruction and infiltration of inflammatory cells and edema in the epithelial layer, mucosa, and submucosa. These histopathological changes were alleviated by AGS administration. The changes in the damage index shown in this results demonstrated that short-term exposure to low pH acid and gastric contents of the esophageal mucosa caused local irritation and inflammation in the esophageal mucosa. 

The acid-induced esophageal mucosal damage is predicted to promote an inflammatory process [37]. We measured the expression level of inflammatory protein to prove the inflammatory and anti-inflammatory response by AGS in damaged esophagus. COX-2 is an inducible enzyme with very low activity in normal tissue cells, but its expression level in inflammatory cells increases sharply, increasing PGE2 content at the inflammation site, resulting in inflammation and tissue damage [40]. The secretion of pro-inflammatory cytokines significantly increased at the site of epithelial injury due to acid reflux [37,41]. Submucosal and epithelial inflammation was associated with increased expression of pro-inflammatory cytokines. Actually, in epithelial immunostaining of acute reflux esophagitis form patients, TNF-α and COX-2 levels showed significant increases in mRNA [38]. In the esophageal mucosa of esophagitis rats, we observed a significant increase in the secretion of COX-2 and TNF-α with increasing chemical damage by acid in the esophageal mucosa. 

The studies of chlorogenic acid and linoleic acid have reported the anti-inflammatory effects by reducing the activation of the NF-κB signaling pathway [42,43,44]. However, research on the NO inhibitory effect of elaidic acid has not been reported. We confirmed the presence of components reported to have anti-inflammatory activity in AGS extracts through HPLC-ELSD and Luna C8, and confirmed the anti-inflammatory activity of components in LPS induced RAW 264.7 cells. The result of the study of molecules in vivo showed a similar tendency to the expression level of the markers confirmed in vitro and to the results of morphological and histopathological analysis. Thus, the degree of damage to the esophageal mucosa is associated with the expression of pro-inflammatory proteins, and AGS is considered to protect the mucosa by inhibiting the inflammatory proteins in the esophageal tissue.

## 5. Conclusions

Our results confirmed the in vitro study of macrophages’ induced inflammation, that AGS has great potential as an anti-inflammatory. The protective effects of AGS were also demonstrated in an esophagitis-induced rat model by regulating the epithelial inflammatory response. Through the results of this study, we illustrate the protective effects of AGS against esophageal injury by regulating expression levels of COX-2 and TNF-α in esophageal tissue. Therefore, we believe that AGS has good physiological activity and can be used as a functional food or resource in the industry for the prevention and therapy of gastroesophageal diseases.

## Data Availability

Not applicable.
